# Phytochemicals and Antioxidant Activity in Oat-Buckwheat Dough and Cookies with Added Spices or Herbs

**DOI:** 10.3390/molecules26082267

**Published:** 2021-04-14

**Authors:** Małgorzata Starowicz, Saruhan Arpaci, Joanna Topolska, Małgorzata Wronkowska

**Affiliations:** Department of Chemistry and Biodynamics of Food, Institute of Animal Reproduction and Food Research of the Polish Academy of Sciences, 10 Tuwima Street, 10-748 Olsztyn, Poland; m.starowicz@pan.olsztyn.pl (M.S.); saruhanarpaci@gmail.com (S.A.); j.topolska@pan.olsztyn.pl (J.T.)

**Keywords:** oat, buckwheat, spice, cookie, phytochemicals, antioxidant activity

## Abstract

The aim of this study was to determine the phytochemicals and antioxidant activity in oat-buckwheat doughs and cookies with the addition of ten selected spices or herbs (2 g/100 g flours weight basis). The used spices and herbs, as was expected, showed a wide range of bioactive molecules, namely phenolic acids and flavonoids, and they are a rich source of components with antioxidant potential. All analysed oat-buckwheat dough showed higher antioxidant activity potential and higher total phenolic content (TPC) compared to cookies. The highest TPC was found in clove, both dough and cookies, with its addition showing the highest ferric reducing antioxidant power. Generally, cookies with the addition of spice/herbs showed higher phytochemical contents and antioxidant activity compared to oat-buckwheat cookies without the condiment. The technology of obtaining confectionery products, like oat-buckwheat cookies, that will favor the protection of bioactive compounds should still be improved.

## 1. Introduction

Oats and buckwheat are already used as raw materials with well-defined nutritional values [1,2]. Oats consumption has recently been boosted due to its various bioactive compounds, which might positively affect human health, such as beta-glucan, antioxidants like avenanthramides, as well as vitamin E (tocotrienols and tocopherols), or phytic acid. All of those compounds are involved in reducing the risk of the development of various diseases like cardiovascular disease, diabetes, obesity, or hypertension, as shown in human interventional studies [1,3].

In turn, buckwheat is a rich source of vitamins, essential amino acids, and phenolic compounds. Health benefits attributed to this pseudocereal include plasma cholesterol level reduction, neuroprotection, anti-inflammatory, and antidiabetic effects, or the alleviation of hypertension conditions [2,4]. Buckwheat can be used within a gluten-free diet because it did not contain protein fractions that are toxic to celiac patients. Oats and buckwheat have already been used as raw materials in the production of different types of bakery products (e.g., bread, cookies) and pasta [5,6].

For centuries, herbs and spices have played an important role as flavoring agents and food preservatives but were also used in folk medicine. According to the International Organization for Standardization [7], spices and condiments are defined as: “Vegetable products or mixtures thereof free from extraneous matter, used for flavouring, seasoning and imparting aroma in foods”. Leaves of a plant are defined as herbs, while spices come from different non-green parts of a plant. Spices and herbs are rich sources of plant phenolics, which may increase the content of ingredients with antioxidant properties, but also retard, e.g., lipid oxidation. Therefore, they can be used as ingredients for functional food formulations [8,9]. Research conducted by Przygodzka et al. [10] proved ethanolic extracts of herbs and spices to be rich sources of phenolics and, resultantly, to present a high antioxidant status. In turn, Mišan et al. [11] showed that ethanolic extracts of different medicinal plants might improve the activity and oxidative stability of cookies. However, the content of spice-derived antioxidants can be decreased after heat treatment [12]. Not many reports have indicated a relationship between the bioactive compounds’ composition of herbs/spices and their functional properties before and after heat treatment. Moreover, the extensive data collected by Rubió et al. [13] in their review article have confirmed that spice-derived compounds are of great interest because of their in vivo and/or in vitro oxidative stress suppression/slowdown and suggested that more efforts need to be undertaken regarding the topic mentioned above.

The enrichment of bakery products with bioactive compounds or compounds with documented impacts on human health can be used as a simple and effective way to improve consumers’ health. Therefore, in this study, we determined the phytochemicals and antioxidant activity in oat-buckwheat cookie formulations with the addition of ten selected spices or herbs.

## 2. Results and Discussion

### 2.1. Total Phenolics Content (TPC)

**Figure 1:**
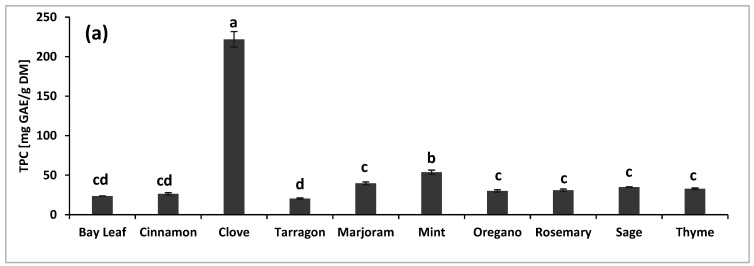

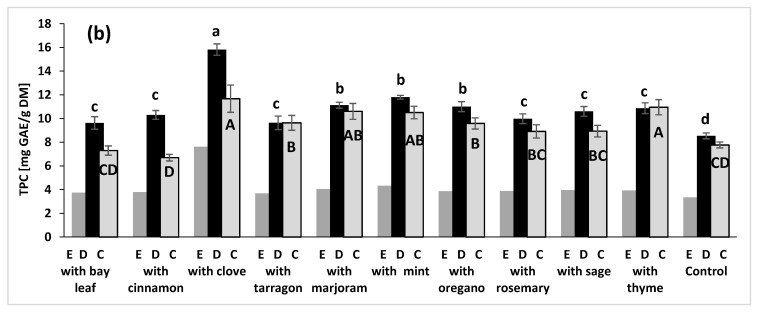
The total phenolic content (TPC) in (**a**) spices/herbs samples and (**b**) doughs—D; cookies—C prepared with spices/herbs, and empiric TPC calculated from its amount in individual components—E. Results are expressed as mean ± standard deviation. Values with different letters (a–d; A–D) are significantly different (*p* < 0.05).

The TPC in ten selected spices or herbs and oat-buckwheat doughs and cookies with their contribution is presented in Figure 1. The highest TPC value was determined in the samples of clove (221.91 ± 9.85 of gallic acid (GAE)/g on a dry matter basis (DM)). The levels of TPC in herbs of the *Lamiaceae* family: marjoram, mint, oregano, rosemary, sage, and thyme, were similar and ranged from 30.02 to 39.67 mg GAE/g DM (Figure 1a). However, almost twice higher TPC was assayed in the mint sample (53.70 ± 2.65 mg GAE/g DM). Significantly (*p* < 0.05) lower TPC values were determined in cinnamon, bay leaf, and tarragon samples. The higher TPC content in clove was also confirmed in the study of Assefa et al. [14]. Moreover, their results showed that TPC was not strongly differentiated among the Lamiaceae species. Our observations following findings reported by Nagy et al. [15], who established that thyme had higher TPC than oregano and sage. The TPC was also determined in flour samples and was almost four-fold higher in buckwheat flour than in oat flour, i.e., 4.48 ± 0.24 and 1.07 ± 0.00 mg GAE/g DM, respectively.

The TPC content in doughs (D) and the cookies (C) were shown in Figure 1b. The empiric content of TPC (E) was calculated from the used dry ingredients TPC content: oat and buckwheat flours and spices or herbs (Figure 1b). The oil used in the recipe does not contain ingredients that could affect the total TPC content. Considering dough analysis, the significantly highest TPC value was determined in the dough with clove addition (Figure 1b). In the case of the other dough samples, their TPC decreased in the following order: dough with mint > marjoram > oregano > thyme > sage > cinnamon > tarragon > bay leaf. The empiric TPC, for all analysed samples, calculated from the used dry ingredients were lower compared to TPC values reported for doughs (Figure 1b). The highest empiric TPC (7.6 mg GAE/g DM) was noticed for the formulation with clove, and the other values were in the range of 3.3–4.3 mg GAE/g DM. In our study, commercial baking powder was used, which is widely used for cookies or muffin production. This is generally a mixture of inorganic compounds (baking soda and acid salts) and starch/flour (added for stability and moisture control). By adding water to this mixture, a chemical reaction is achieved, producing carbon dioxide, which is trapped in tiny air pockets in the dough or batter [16]. Carullo et al. [16] showed the possibility of using a baking powder as the carrier of ingredients with antioxidant properties.

The baking process (185 °C for 20 min) used in our study caused a significant decrease in the content of TPC in the obtained cookies compared to the dough. A decrease in TPC ranging from 11% to 15% was noted in the cookie samples with mint, oregano, rosemary, and sage compared to the dough. Also, it should be noted that buckwheat flour (TPC—4.48 mg GAE/g DM) significantly contributed to the TPC values of cookies. The value noted for the control cookie (TPC—7.77 mg GAE/g DM,) was similar to this presented by Jan et al. [17], who investigated incorporating 40%–60% of buckwheat flour into a gluten-free formulation. As was mentioned by Li et al. [18] and Rosales-Soto et al. [19] baking could reduce the TPC in muffins.

### 2.2. Determination of Bioactive Compounds by HPLC-MS/MS

The results of TPC measured with the Folin-Ciocalteu procedure failed to give a full picture of the quality or even quantity of the phenolics composition in the extracts. Therefore, the next stage of this study involved determinations of the contents of the main phytochemicals for checking which specific compounds might most affect the antioxidant properties analysed in this study. The contents of phenolic acids and flavonoids were determined with the high-pressure liquid chromatography-tandem mass spectrometry (HPLC-MS/MS) method and collected in Table 1 and Table 2. Six phenolic acids (ferulic, sinapic, *p*-coumaric, rosmarinic, chlorogenic, and caffeic) were identified in the samples of spices and herbs, whereas vanillic acid was found only in rosemary (Table 1). The highest total content of phenolics, presented as a sum of individual phenolic acids, was found in sage (100.46 µg/100 g DM), followed by marjoram (87.15 µg/100 g DM), thyme (81.35 µg/100 g DM), and rosemary (73.58 µg/100 g DM). These investigations confirmed rosmarinic acid presence in cinnamon. This acid is the major antioxidant compound in the Lamiaceae herbs, as pointed out by Wojdyło et al. [20]. Despite that, it wasn’t detected in the sample of cinnamon studied by Lu et al. [21], presumably due to the use of a less sensitive HPLC-MS/MS technique, nor in the samples of thyme, sage, and oregano analysed by Nagy et al. [15]. In the present study, the highest content of rosmarinic acid was found in marjoram (83.76 µg/100 g DM), which accounted for almost 96% of the TPC. Rosmarinic acid was not detected only in the clove. In turn, chlorogenic acid turned out to be the major bioactive compound of this spice. The second most abundant phenolic compound in spices and herbs was caffeic acid, with its content ranging from 0.11 to 7.57 µg/100 g DM. This was an important finding because caffeic acid is an essential bioactive compound considering its high activity as an antioxidant [22]. The lowest contents of phenolic compounds were found in bay leaf and cinnamon. Raguindin et al. [23] in the review presented that the total content of phenolic in oat products is ranged from 180 to 576 mg rutin equivalent/100 g and the most common compound found in the literature was ferulic acid (max. 148.36 mg/100 g). While for the buckwheat products the same authors showed the total content of phenolics reported in the literature is 123–3149 mg rutin equivalent/100 g, with the most common p-anisic acid (max. 1190 mg/100 g). In our study, in oat flour, only protocatechuic acid was detected (2.17 µg/g DM), while in buckwheat flour none of analysed phenolic acids was found.

Four molecules of phenolic acids were detected in the cookies with an addition of selected spices or herbs: ferulic, rosmarinic, chlorogenic, and caffeic acids (Table 1). This means that the heat treatment had a negative impact on the structure of sinapic, *p*-coumaric, and vanillic acids. In the analysed cookies, the total content of phenolics ranged from 0.05 (cookies with bay leaf) to 2.31 µg/100 g DW (cookies with tarragon). As expected, the highest content of rosmarinic acid was established in all cookies. It accounted for 58–93% of total phenolic compounds in the analysed cookies. The cookies with tarragon had a high content of chlorogenic acid (91.59 µg/g DM), which was higher than in the gluten-free bread incorporated with coffee husk (20 µg/g DM) but lower than in the bread with coffee silverskin addition (250 µg/g DM) [24]. Moreover, only rosmarinic and caffeic acids were detected in the control cookies, which might suggest that ferulic and chlorogenic acids were incorporated into cookies directly from spices or herbs.

Seven predominant flavonoids were identified in spices and herbs: apigenin, rutin, catechin, naringenin, luteolin, vitexin, and kaempferol (Table 2). The highest total flavonoid content, presented as a sum of individual flavonoid compounds, was noted in mint (9.71 µg/100 g DM), followed by bay leaf (7.66 µg/100 g DM), then marjoram (5.59 µg/100 g DM). Cinnamon had the lowest content of phenolics and also flavonoids. Apart from catechin and naringenin, no other flavonoid compounds were found in cinnamon samples. Catechin was determined in bay leaf, cinnamon, clove, and mint as the only representative of the *Lamiaceae* family. The major flavonoid substances in the tested spice/herbs samples were luteolin, naringenin, and apigenin. Obtained results are consistent with findings reported by Wojdyło et al. [20], who identified luteolin and apigenin as the major flavonoids in sage and rosemary. Moreover, vitexin was determined only in bay leaf, marjoram, and thyme. Vitexin accounted for 50% and 58% of the total flavonoid content in bay leaf and marjoram, respectively. Furthermore, few flavonoids were determined in the cookies, which were found to contain rutin, catechin, naringenin, luteolin, (Table 2). The content of rutin was similar in all cookie samples (around 0.02 µg/g DM). This may be explained by the fact that rutin is the most abundant bioactive compound in buckwheat [2], while buckwheat flour content in cookies formula was at 50%. The highest content of catechin was found in the cookies with clove (15.19 µg/g DM), mint, and bay leaf (approx. 12 µg/g DM). Naringenin was determined in negligible amounts in the cookies with rosemary and sage. The main flavonoids found in the buckwheat product were rutin (max. 5186 mg/100 g) and quercetin (max. 857.62 mg/100 g) as presented by Raguindin et al. [23], and rutin (0.247 µ/g) was found in buckwheat flour used in our study. In oat flour, none of the analysed flavonoids were detected.

### 2.3. Determination of Reducing Potential and Antioxidant Activity

The reducing potential of the ethanolic extracts of the analysed plant material and prepared dough/cookies were measured by the ferric reducing antioxidant power (FRAP) assay, and the results are shown in Table 3. According to the results obtained for spice/herb extracts, the clove (508.77 µmol Trolox/g DM) exhibited a higher ability to reduce Fe(III) to Fe(II), followed by rosemary (469.55 µmol Trolox/g DM). For these herbs, the Trolox equivalents were significantly higher than for the other extracts (*p* < 0.05). In the study of Lu et al. [21], the FRAP values of cinnamon and bay leaf samples were 50% higher than in this study, which might be due to different extraction procedures applied. Nevertheless, the average FRAP value was estimated for spices and herbs at 290.2 µmol/g [25], which is twice lower than in clove and rosemary samples in this study. The FRAP values obtained for the flours used in the recipes were: 8.50 ± 0.08 for oat flour and 19.71 ± 0.15 µmol Trolox/g DM for buckwheat flour.

Compared to the other analysed doughs, the ferric-reducing antioxidant power of the control dough, prepared from oat and buckwheat flour, sugar, baking powder, rapeseed oil, egg, and water, was the lowest and reached 6.50 µmol Trolox/g DM (Table 3). The highest value was noticed for the dough with clove (137.01 µmol Trolox/g DM), whereas for the other dough samples with spices/herbs this value was at about 100 µmol Trolox/g DM. The FRAP values determined for all analysed doughs were from 3 to 5-times lower compared to spices/herbs extracts. The baking process at 185 °C/20 min caused a significant reduction in the FRAP values of cookies (Table 3). Also, for all cookies with spices/herbs, the obtained results were more than 10-times lower compared to doughs. The FRAP value determined for the control cookies was in accordance with results found for the gluten-free bread with 40%–60% buckwheat addition [17], and slightly higher than the average FRAP value reported for snacks and biscuits (5.8 µmol/g) by Carlsen et al. [25]. Compared to the control sample for cookies with clove, with marjoram, mint, and thyme a significant increase in reducing potential was observed.

The ability of antioxidants from all analysed extracts to scavenge superoxide anion radicals (O_2_^−•^) was determined with the photochemiluminescence (PCL) method. The data obtained for water-soluble (ACW) and lipid-soluble (ACL) antioxidants, and their sum are presented in Table 4. The results of ACW, ACL, and their sum (PCL = ACW + ACL) obtained for flours used in this study were: 61.67 ± 1.85, 5.14 ± 0.17, and 66.81 µmol Trolox/g DM for oat flour; and 122.54 ± 24.93, 15.93 ± 0.78, and 138.47 µmol Trolox/g DM for buckwheat flour. Similarly, it was observed for reducing potential, extracts from spices/herbs had a significantly higher antioxidant activity compared to doughs and cookies. Among the analysed spice/herbs bay leaf, cinnamon, tarragon, and marjoram had a higher value of ACW than ACL, while the values of ACL were higher in the other analysed spices/herbs. Control dough and cookies had almost 2-times higher values of ACL compared to ACW. However, when spices/herbs were used in dough and cookies, no such significant differences were found in the ACW and ACL values. Cookies with thyme, mint, rosemary, and sage were characterized by the highest antioxidant activity measured as a sum of ACW and ACL.

Due to the increasing interest in the health-promoting properties of food, antioxidants are in the focus of interest of researchers and consumers. Some epidemiological surveys have shown certain health benefits to be positively correlated with the consumption of plant-derived foods [26]. However, noteworthy is the “antioxidant paradox” resulting from human intervention studies, which failed to demonstrate the preventive or therapeutic effect of large doses of dietary antioxidant supplementation [27,28]. Assefa et al. [14] showed that among 39 spices/herbs, the five spices with the most antioxidants were: cloves > allspice > cinnamon > oregano > marjoram. In this study, high content of antioxidants was confirmed in clove (3141.1 µmol Trolox/g DM), and it resulted in the high content of antioxidants in the dough (42.6 µmol Trolox/g DM) and cookies with clove addition (27.3 µmol Trolox/g DM). Arashahi-D et al. [29] showed that the antioxidant activity of extracts from mint leaves increased significantly, by about 10%, after boiling. A similar observation was made by Shobana et al. [30], according to whom the antioxidant activity of ginger, garlic, onion, mint leaves, cinnamon, cloves, and pepper was retained after boiling at 100 °C for 30 min.

### 2.4. Correlation Study

Total phenolics content and antioxidant activity determined in the samples of spices and herbs showed a good correlation with: TPC vs. FRAP (*R* = 0.622), TPC vs. PCL (*R* = 0.680), TPC vs. ACL (*R* = 0.745). Values of correlation coefficients obtained in this study were lower than those demonstrated by Wojdyło et al. [20]. This is might be due to not focusing on one family of herbs and spices in this study. Therefore, high correlation coefficients were calculated for TPC vs. sinapic acid contents (*R* = 0.939). It might indicate that these compounds mostly contributed to the total content of polyphenols in spice/herb samples. Moreover, a significant and positive correlation was noted between the content of phenolics (presented as a sum of individual compounds) and caffeic, and rosmarinic acids, i.e., 0.893 and 0.990, respectively. Although not high, a positive correlation was also found among flavonoids and rutin (*R* = 0.656), and luteolin contents (*R* = 0.690). The coefficients of correlation with the antioxidant activity measured with the PCL method were: *R* = 0.722 between PCL and sinapic acid, *R* = 0.645 between PCL and FRAP, and *R* = 0.680 between PCL and ACL. A significant linear correlation was found between ACL and sinapic acid (*R* = 0.697). It suggests that lipid-soluble antioxidants play an important role in increasing the antioxidant potential of aromatic plant material. These findings are consistent with the results of a study conducted by Lagouri and Boskou [31], who found a significant correlation between the content of essential oils from spices and their high antioxidant activity. Thus, high values of the correlation coefficient were noted between both methods used to measure the reducing and antioxidant potential, ACL, and FRAP (*R* = 0.812). Interestingly, the relationship between the contents of catechin and vitexin, and sinapic acid and kaempferol, was found to be positively correlated (*R* = 0.787 and 0.883, respectively). It seems that the formation of these particular biologically active compounds does not follow a competition mechanism. These observations do not explain a complex mechanism of bioactive compound formation, which might however be linked to the shikimate biosynthesis pathway of phenolic compounds in plant tissues and its possible modifications by biotic and abiotic stress [32].

In dough, a positive correlation between total phenolics content and antioxidant activity was demonstrated as TPC vs. FRAP (*R* = 0.700), TPC vs. PCL (*R* = 0.704), TPC vs. ACL (*R* = 0.764), and TPC vs. ACW (*R* = 0.642). According to a previous study concerning selected spices fortified rye-buckwheat cakes, TPC vs. ACW was at the same level [33]. Furthermore, significantly higher values of correlation coefficients were found in this study among ACW and FRAP (*R* = 0.801). The antioxidant activity of dough samples was strongly correlated with water- and lipid-soluble antioxidants as ACW vs. ACL (*R* = 0.832), then ACW vs. PCL (*R* = 0.985), and ACL vs. PCL (*R* = 0.912). The same correlations were also observed in cookie samples: ACW and ACL (*R* = 0.718), ACW and PCL (*R* = 0.939), and ACL and PCL (*R* = 0.913). Also, a strong correlation was noticed in cookies between results of the following assays: FRAP and ACW (*R* = 0.829), and FRAP and PCL (*R* = 0.723). Particularly high correlation coefficients were noted between the antioxidant activity and contents of individual compounds: ACL and rosmarinic acid, ACL vs. caffeic acid; PCL and caffeic acid, *R* = 0.771; 0.882; 0.756, respectively. In this case, it may suggest that rosmarinic and caffeic acids take part in the formation of high overall antioxidant activity of cookies with spices/herbs addition. Surprisingly, neither in this study nor in the previous one [33], where did not find any strong correlation between rutin content (which is a major flavonoid in buckwheat flour) and antioxidant activity of cookies prepared with buckwheat blends. However, for all cookies, the sum of certain phenolic compounds was in strong correlation with rosmarinic acid (*R* = 0.961), and caffeic acid (*R* = 0.812). In addition, chlorogenic acid content correlated significantly with naringenin and ferulic acid contents (*R* = 0.767; 0.846). The high correlation coefficients were also determined between flavonoid compounds as luteolin vs. rutin (*R* = 0.905), and phenolic acids like rosmarinic vs. caffeic (*R* = 0.917) for all cookies.

## 3. Materials and Methods

### 3.1. Chemicals

Aluminum chloride hexahydrate (AlCl_3_∙6H_2_O); 6-hydroxy-2,5,7,8-tetramethylchromane-2-carboxylic acid (Trolox); Folin-Ciocalteu’s phenol reagent; 2,4,6-tris(2-pyridyl)-1,3,5-triazine (TPTZ); iron (III) chloride hexahydrate (FeCl_3_∙6H_2_O); sodium acetate; acetic and hydrochloric acids; gallic acid; water and acetonitrile (both of MS grade); formic acid and diethyl ether were purchased from Sigma Chemical Co. (St. Louis, MO, USA). PCL kits for water-soluble (ACW) and lipid-soluble (ACL) antioxidants were bought from Analytik Jena (Jena, Germany). Standards of phenolic acids and flavonoids as apigenin, caffeic acid, catechin, chlorogenic acid, kaempferol, ferulic acid, isorhamnetin acid, luteolin, naringenin, sinapic acid, *p*-coumaric acid, protocatechuic acid, quercetin, rosmarinic acid, rutin, vanillic acid, and vitexin were supplied from Sigma Chemical (St. Louis, MO, USA) and Extrasyntese (Genay, Lyon area, France). Water was purified using a Mili-Q-system (Millipore, Bedford, MA, USA).

### 3.2. Materials

Commercial buckwheat (*Fagopyrum esculentum* Moench) and oats (*Avena sativa* L.) flours were purchased from a local flour production plant (Melvit S.A., Kruki, Poland). As declared by the producer, contents of carbohydrates, protein, ash, fat, and fiber in buckwheat flour on dry matter (DM) basis were: 65.2%; 19.2%; 3.2%; 0.7%, and 2.3%, respectively, and in oat flours were: 60.4%; 15.4%; 2.1%; 7.1%; and 9.7% respectively.

The spices and herbs available on the market, including *Laurus nobilis* L. (Lauraceae; bay leaf), bark of *Cinnamomum verum* J.Presl (Lauraceae; cinnamon), flower buds of *Syzygium aromaticum* (L.) Merr. & L.M.Prry (Myrtaceae; clove), *Artemisia dracunculus* L. (Asteraceae; tarragon), and representatives of the *Lamiaceae* Lindl. family: *Origanum majorana* L. (marjoram), *Mentha* aquatica L. (mint), *Origanum vulgare* L. (oregano), *Rosmarinus officinalis* L. (rosemary.), *Salvia aegyptiaca* L. (sage.), and *Thymus vulgaris* L. (thyme) (www.ziolowyzakatek.sklep.pl, accessed on 31 October 2018, Poland) were used in this study. These ten spices or herbs were milled into powder to particles <400 μm before addition to cookies (mill WZ-1, ZBPP, Bydgoszcz, Poland).

### 3.3. Cookies Preparation

Experimental recipes were based on 100 g of oat flour, 100 g of buckwheat flour, 25 g of sugar, 3 g of baking powder, 70 g of rapeseed oil, 68 g of egg, 45 mL of tap water, and 4 g of selected spices or herbs (2 g/100 g flours weight basis). All dry ingredients were mixed and then oil, egg, and water were added and mixed for 3 min (Kitchen Aid, St. Joseph, MI, USA). The dough was cut with a square cookie cutter (60 mm) and part of the dough was freeze-dried. An electric oven (DC-21 model, Sveba Dahlen AB, Fristad, Sweden) was used to bake the cookies (185 °C for 20 min). Obtained cookies were freeze-dried, then dry samples were milled, and stored in a refrigerator (−70 °C) until analyses. The addition of spices/herbs to the cookie formulas was selected based on the results of preliminary sensory tests (data not showed). Also, baking parameters were established according to the previous experiments and initial trials to achieve the highest sensory acceptability (data not showed).

### 3.4. Extraction Procedure

Briefly, the flours, cookies, or spices/herbs sample (100 mg) was extracted with 1 mL of 50% ethanol. The type of solvent used during extraction has been chosen according to previous research [10]. The mixture was vortexed and sonicated for 30 s (VC 750, Sonics & Materials, Newtown, CT, USA). This procedure was repeated 3 times, followed by centrifugation at 14,000× *g* and 4 °C for 10 min (Centrifuge 5425R, Eppendorf, Hamburg, Germany). After centrifugation, the supernatant was collected into a 5-mL volumetric flask. This procedure was repeated 5 times to obtain 5 mL of the extracts. The extracts were then kept in a freezer (−20 °C) until further analysis, including the determination of total phenolics content (TPC), antioxidant activity, and reducing potential (PCL ACW and ACL, and FRAP), and content of phytochemicals. Before each analysis, extracts were filtered through filters (Whatman, Marlborough, MA, USA) to remove any remaining particles.

### 3.5. Determination of Total Phenolic Content (TPC)

The TPC method was adopted on microplates according to the procedure described by Horszwald and Andlauer [34]. The appropriate extract (15 µL) was placed into a well (96-well microplate, transparent, Porvair, Wrexham, UK). Then, 240 µL of Folin-Ciocalteu phenol reagent (diluted with water at 1:15, *v*/*v*) was added. The mixture was incubated in the dark at room temperature for 10 min. Then, 15 µL of 20% sodium carbonate (Na_2_CO_3_) was added to each well. The microplate was shaken for 30 s before analysis and the absorbance was measured at λ = 755 nm using an Infinite M1000 microplate reader (Tecan, Männedorf, Switzerland). The 50% ethanol solution was used as a blank, and gallic acid was used as a standard to plot the calibration curve. The stock solution of gallic acid (1 mg/mL) was prepared in 50% ethanol. The calibration was prepared with a few dilutions of gallic acid (GAE).

### 3.6. Determination of Contents of Phenolic Acids and Flavonoids Using High-Pressure Liquid Chromatography-Tandem Mass Spectrometry (HPLC-MS/MS)

The profile and content of phenolic acids and flavonoids were determined according to the modified method of Wiczkowski et al. [35]. In this case, 1 mL of 50% extracts was evaporated to dryness under nitrogen at 35 °C. Then, all samples with dry residue were dissolved in 0.1 mL of 80% methanol, centrifuged (13,200× *g* at 4 °C, 20 min), and used for the HPLC-MS analysis. Aliquots (2 µL) of extracts were injected into an HPLC system (LC-200, Eksigent, Framingham, MA, USA) equipped with a dual-channel pump, a column oven, an autosampler (set at 4 °C), and a system controller linked to the Analyst 1.5.1 system. Chromatographic separation was conducted with a HALO C18 column (2.7 µm particles, 0.5 × 50 mm, Eksigent, Framingham, MA, USA) at 45 °C and a flow rate of 15 µL/min. The elution solvents used were: A (water/formic acid; 99.05/0.95; *v*/*v*) and B (acetonitrile/formic acid, 99.05/0.95, *v*/*v*). The gradient was used as follows: 5% B for 0.1 min, 5–90% B in 1.9 min, 90% B for 0.5 min, 90–5% B in 0.2 min, and 5% B for 0.3 min. For HPLC-MS/MS analysis, a QTRAP 5500 ion trap mass spectrometer (AB SCIEX, Framingham, MA, USA) was connected to the Eksigent LC200 via an ESI interface. Optimal ESI-MS/MS conditions including nitrogen curtain gas, collision gas, ion spray source voltage, temperature, nebulizer gas, and turbo gas were as follows: 25 L/min, 9 L/min, −4500 V, 350 °C, 35 L/min, and 30 L/min, respectively. Qualitative and quantitative analyses were made using the multiple reaction monitoring (MRM) method for appropriate external standards.

### 3.7. Determination of Reducing Potential by the Ferric Reducing Power (FRAP) Assay and Antioxidant Activity Using the Photochemiluminescence (PCL) Method

The ferric reducing power was determined using the FRAP assay, based on the reduction of ferric ion by antioxidant compounds, according to Horszwald and Andlauer [34]. The absorbance of the mixture was measured at 593 nm after a 5-min reaction with the microplate reader (M1000 Infinite, Tecan, Switzerland). All measurements were conducted in triplicate. The results obtained were expressed as µmol Trolox per gram of the DM sample.

The photochemiluminescence (PCL) method was used to measure the ability of antioxidants from specific extracts to scavenge superoxide anion radicals (O_2_^−•^). The measurement was performed using the PHOTOCHEM^®^ apparatus (Analytik Jena, Jena, Germany) according to the protocols elaborated by Zieliński et al. [36]. The 50% ethanolic extracts were determined with two methodologies for water-soluble (ACW) and lipid-soluble (ACL) antioxidants. The results were expressed as µmol Trolox per gram of the sample, whereas the total PCL values were calculated as the sum of the results obtained for ACL and ACW. All measurements were conducted in triplicate. The results obtained were expressed as µmol Trolox per gram of the DM sample.

### 3.8. Statistical Analysis

All analyses were performed in triplicate, and the average values with standard deviations (AV ± SD) were reported. The analysis of variance (ANOVA) followed by Tukey’s multiple comparison test was used. Statistical analysis software system GraphPad Prism version 8.0.0 for Windows (GraphPad Software, San Diego, CA, USA) was used for analyses. Differences between mean values were found to be significant at *p* < 0.05.

## 4. Conclusions

Results showed that spices and herbs, including bay leaf, cinnamon, clove, tarragon, marjoram, mint, oregano, rosemary, sage, and thyme, were rich sources of bioactive compounds and demonstrated high antioxidant activity measured with various methods. Moreover, the qualitative and quantitative analysis of phenolic and flavonoid compounds helped explain which of the compounds had a significant influence on the antioxidant activity in spices and herbs. In the tested spice/herbs samples, the phenolic acids: rosmarinic, caffeic, and chlorogenic constituted the largest quantitatively group, while, the major flavonoid substances were: luteolin, naringenin, and apigenin. The obtained cookies did not show such a high content of TPC, phenolic acids, or flavonoids compared to spices/herbs. However, it should be noted that, compared to control, cookie samples with the addition of spice/herbs showed higher phytochemical contents and antioxidant activity. Further research should concern the modification of the technological process used for oat-buckwheat cookies preparation to maintain the greatest possible number of beneficial properties derived from the ingredients used in the recipe.

## Figures and Tables

**Table 1:** Content of phenolic acids in 80% methanolic extracts of the analysed spices/herbs, and cookies with their contribution. The total content of phenolic compounds in each sample is presented in the last column of the table. Data presented as µg/g of DM; nd—non detected.

Sample	Ferulic Acid	Sinapic Acid	*p*-Coumaric Acid	Rosmarinic Acid	Chlorogenic Acid	Vanillic Acid	Caffeic Acid	Total
**Spices/herbs**								
bay leaf	nd	nd	6.49 ± 0.02 ^e^	8.18 ± 0.45 ^e^	8.41 ± 0.25d ^e^	nd	nd	23.08
cinnamon	nd	nd	nd	8.39 ± 0.58 ^e^	nd	nd	nd	8.39
clove	12.59 ± 1.05 ^b^	20.17 ± 2.24 ^a^	35.47 ± 0.79 ^b^	nd	291.20 ± 15.67 ^b^	nd	10.95 ± 0.65 ^e^	370.38
tarragon	51.67 ± 3.48 ^a^	4.60 ± 0.57 ^b^	16.71 ± 1.53 ^c^	1855.01 ± 121.29 ^d^	789.94 ± 89.61 ^a^	nd	101.47 ± 5.84 ^d,e^	2819.40
marjoram	nd	nd	16.27 ± 1.88 ^c^	8376.82 ± 364.39 ^a^	57.99 ± 4.79 ^d^	nd	263.49 ± 26.36 ^c^	8714.57
mint	7.11 ± 0.58 ^c^	1.04 ± 0.04 ^c^	11.42 ± 0.31 ^d^	5314.19 ± 110.39 ^c^	55.81 ± 1.31 ^d^	nd	180.57 ± 1.55 ^c,d^	5570.14
oregano	nd	2.01 ± 0.08 ^bc^	12.56 ± 0.35 ^d^	3454.52 ± 223.56 ^c^	155.22 ± 13.07 ^c^	nd	147.31 ± 5.77 ^c,d^	3771.62
rosemary	1.78 ± 0.13 ^d^	3.47 ± 0.16 ^bc^	13.34 ± 1.09 ^d^	6715.85 ± 604.95 ^b^	71.07 ± 0.14 ^d^	44.21 ± 2.98	508.66 ± 35.12 ^b^	7358.38
sage	2.30 ± 0.12 ^d^	nd	1364.00 ± 0.44 ^a^	7914.83 ± 74.30 ^a^	7.34 ± 1.33d ^e^	nd	757.39 ± 72.81 ^a^	10,045.86
thyme	1.29 ± 0.14 ^d^	nd	9.13 ± 0.21 ^d,e^	7528.76 ± 98.37 ^a^	41.73 ± 2.01 ^d^	nd	554.50 ± 39.25 ^b^	8135.41
**Cookies with**								
bay leaf	nd	nd	nd	5.15 ± 0.12 ^e^	nd	nd	nd	5.15
cinnamon	nd	nd	nd	nd	nd	nd	nd	-
clove	0.29 ± 0.02 ^b^	nd	nd	nd	6.84 ± 0.25 ^b^	nd	nd	7.13
tarragon	0.63 ± 0.10 ^a^	nd	nd	132.91 ± 14.94 ^c^	91.59 ± 5.62 ^a^	nd	5.87 ± 0.96 ^d^	231.00
marjoram	nd	nd	nd	174.55 ± 6.54 ^b^	1.25 ± 0.07 ^b,c^	nd	12.10 ± 0.47 ^c^	187.90
mint	0.21 ± 0.01 ^b^	nd	nd	151.82 ± 5.18 ^c^	1.90 ± 0.20 ^b,c^	nd	14.27 ± 0.31 ^b^	168.20
oregano	nd	nd	nd	103.18 ± 7.25 ^d^	2.60 ± 0.11 ^b,c^	nd	7.28 ± 0.28 ^d^	113.06
rosemary	0.25 ± 0.07 ^b^	nd	nd	107.24 ± 4.28 ^d^	1.51 ± 0.03 ^b,c^	nd	13.25 ± 0.26 ^b,c^	122.25
sage	nd	nd	nd	196.50 ± 7.17 ^a^	1.37 ± 0.21 ^b,c^	nd	19.46 ± 0.89 ^a^	217.33
thyme	nd	nd	nd	129.19 ± 5.86 ^c^	1.92 ± 0.04 ^b,c^	nd	12.48 ± 0.88 ^c^	143.59
control	nd	nd	nd	6.63 ± 0.37 ^e^	nd	nd	2.98 ± 0.32 ^e^	9.61

Results are expressed as mean ± standard deviation. Values in the same column followed by different superscript letters are significantly different (*p* < 0.05).

**Table 2:** Content of flavonoid compounds in 80% methanolic extracts of the analysed spices/herbs and extracts of cookies with their contribution. Data presented as µg/g of DM; nd—non detected.

Sample	Apigenin	Rutin	Catechin	Naringenin	Luteolin	Vitexin	Kaempferol	Total
**Spices/herbs**								
bay leaf	nd	0.28 ± 0.04 ^c^	315.85 ± 18.81 ^a^	1.19 ± 0.04 ^f^	nd	444.55 ± 27.47 ^a^	4.59 ± 0.20 ^d^	766.46
cinnamon	nd	nd	39.15 ± 1.98 ^b^	0.71 ± 0.03 ^f^	nd	nd	nd	39.86
clove	nd	0.03 ± 0.00 ^e^	37.69 ± 1.17 ^b^	14.47 ± 0.34 ^e^	137.26 ± 5.62 ^c^	nd	30.54 ± 2.18 ^a^	219.99
tarragon	5.48 ± 0.09 ^c^	0.42 ± 0.04 ^b^	nd	93.82 ± 6.52 ^b^	20.76 ± 0.20 ^e^	nd	17.75 ± 2.47 ^b^	138.23
marjoram	13.54 ± 0.92 ^b^	0.05 ± 0.00 ^e^	nd	24.98 ± 3.37 ^d^	236.57 ± 8.61 ^b^	277.90 ± 14.48 ^b^	6.43 ± 0.40 ^c^	559.47
mint	15.19 ± 0.91 ^a,b^	0.64 ± 0.04 ^a^	23.88 ± 1.21 ^b^	20.18 ± 2.32 ^d,e^	903.24 ± 35.13 ^a^	nd	7.73 ± 0.12 ^c^	970.86
oregano	15.82 ± 0.78 ^a^	0.10 ± 0.00 ^d,e^	nd	73.71 ± 6.62 ^c^	123.18 ± 4.31 ^c^	nd	9.31 ± 0.26 ^c^	222.12
rosemary	7.09 ± 0.10 ^c^	0.15 ± 0.02 ^d^	nd	0.93 ± 0.03 ^f^	197.46 ± 20.96 ^b^	nd	nd	205.63
sage	3.03 ± 0.25 ^d^	0.03 ± 0.00 ^e^	nd	0.77 ± 0.05 ^f^	107.77 ± 18.44 ^c^	nd	nd	111.6
thyme	4.75 ± 0.30 ^c^	0.02 ± 0.00 ^e^	nd	109.22 ± 4.12 ^a^	84.96 ± 1.88 ^d^	67.66 ± 3.51 ^c^	3.56 ± 0.36 ^d^	270.17
**Cookies with**								
bay leaf	nd	0.01 ± 0.00	11.69 ± 1.40 ^a^	0.55 ± 0.03 ^cd^	nd	nd	nd	12.25
cinnamon	nd	0.02 ± 0.00	9.70 ± 0.28 ^b^	0.29 ± 0.02 ^d^	nd	nd	nd	10.01
clove	nd	0.02 ± 0.00	15.19 ± 0.93 ^a^	0.27 ± 0.04 ^d^	1.53 ± 0.17 ^d^	nd	nd	17.01
tarragon	nd	0.02 ± 0.00	2.67 ± 0.14 ^b,c^	2.54 ± 0.45 ^a^	nd	nd	nd	11.09
marjoram	nd	0.02 ± 0.00	1.10 ± 0.56 ^c^	0.55 ± 0.05 ^c,d^	5.32 ± 0.35 ^b^	nd	nd	16.88
mint	nd	0.05 ± 0.00	12.96 ± 0.47 ^a^	0.78 ± 0.09 ^c^	36.60 ± 0.90 ^a^	nd	nd	50.39
oregano	nd	0.02 ± 0.00	1.41 ± 0.43 ^c^	1.41 ± 0.03 ^b^	3.42 ± 0.05 ^c^	nd	nd	6.26
rosemary	nd	0.02 ± 0.00	1.38 ± 0.24 ^c^	nd	3.37 ± 0.04 ^c^	nd	nd	4.77
sage	nd	0.03 ± 0.00	1.10 ± 0.07 ^c^	nd	2.21 ± 0.05 ^d^	nd	nd	3.35
thyme	nd	0.02 ± 0.00	nd	1.44 ± 0.02 ^b^	1.60 ± 0.06 ^d^	nd	nd	3.06
control	nd	0.02 ± 0.00	9.19 ± 0.10 ^b^	nd	nd	nd	nd	9.21

Results are expressed as mean ± standard deviation. Values in the same column followed by different superscript letters are significantly different (*p* < 0.05).

**Table 3:** Antioxidant activity measured with the FRAP method. Data presented as µmol Trolox/g DM.

Sample	FRAP		
	Spices/Herbs	Doughs	Cookies
bay leaf	244.64 ± 2.85 ^d,e^	92.63 ± 3.84 ^c^	7.30 ± 0.39 ^c,d^
cinnamon	220.76 ± 11.14 ^e^	92.19 ± 2.33 ^c^	6.70 ± 0.28 ^d^
clove	508.77 ± 8.57 ^a^	137.01 ± 2.38 ^a^	11.68 ± 1.15 ^a^
tarragon	374.44 ± 0.53 ^b^	92.93 ± 3.26 ^c^	9.64 ± 0.63 ^b^
marjoram	293.27 ± 5.87 ^c^	100.91 ± 2.80 ^c,b^	10.61 ± 0.67 ^a,b^
mint	378.76 ± 9.31 ^b^	108.79 ± 5.72 ^b^	10.51 ± 0.53 ^a,b^
oregano	331.68 ± 7.14 ^c^	93.55 ± 0.99 ^c^	9.59 ± 0.47 ^b^
rosemary	469.55 ± 37.06 ^a^	99.56 ± 2.50 ^c^	8.92 ± 0.56 ^bc^
sage	392.18 ± 13.95 ^b^	95.39 ± 1.32 ^c^	8.94 ± 0.49 ^b,c^
thyme	284.77 ± 17.89 ^d^	98.34 ± 2.44 ^c^	10.96 ± 0.64 ^a^
control	-	6.50 ± 0.73 ^d^	7.77 ± 0.25 ^c,d^

Results are expressed as mean ± standard deviation. Values in the same column followed by different superscript letters are significantly different (*p* < 0.05).

**Table 4:** Antioxidant activity measured with the PCL method for water-soluble (ACW) and lipid-soluble (ACL) antioxidants in herbs/spices, doughs, and cookies with their contribution. The sum of ACW and ACL is presented as PCL. Data presented as µmol Trolox/g DM.

Sample	ACW			ACL			PCL		
	Spices/Herbs	Doughs	Cookies	Spices/Herbs	Doughs	Cookies	Spices/Herbs	Doughs	Cookies
bay leaf	1741.09 ± 132.78 ^a^	7.91 ± 0.33 ^d^	4.02 ± 0.17 ^c^	385.61 ± 7.80 ^g^	14.34 ± 0.64 ^c^	9.35 ± 0.40 ^g^	2126.7	22.25	13.37
cinnamon	732.08 ± 24.00 ^c^	7.15 ± 0.39 ^d^	1.86 ± 0.08 ^c^	259.26 ± 3.03 ^h^	13.20 ± 0.38 ^c^	9.12 ± 0.38 ^g^	991.34	20.35	10.98
clove	873.00 ± 35.98 ^c^	21.98 ± 0.59 ^b^	15.60 ± 3.38 ^a^	2268.13 ± 62.96 ^a^	20.62 ± 0.72 ^a^	11.69 ± 0.41 ^f^	3141.13	42.60	27.29
tarragon	993.16 ± 60.82 ^b^	14.28 ± 3.84 ^c^	4.89 ± 0.12 ^c^	454.01 ± 21.93 ^g^	13.78 ± 0.69 ^c^	12.70 ± 0.45 ^e^	1447.17	28.06	17.59
marjoram	865.51 ± 55.60 ^bc^	17.98 ± 1.93 ^bc^	8.55 ± 0.32 ^b^	784.96 ± 24.45 ^f^	17.48 ± 0.64 ^b^	15.40 ± 0.43 ^d^	1650.47	35.46	23.95
mint	821.90 ± 38.18 ^c^	27.81 ± 1.86 ^a^	14.59 ± 0.26 ^a^	1018.38 ± 37.20 ^e^	18.25 ± 1.19 ^b^	19.86 ± 0.19 ^a,b^	1840.28	46.06	34.45
oregano	1097.30 ± 70.56 ^b^	14.09 ± 1.08 ^c^	10.50 ± 0.15 ^b^	1307.33 ± 16.96 ^c^	15.60 ± 0.76 ^c^	17.46 ± 0.93 ^c^	2490.26	29.69	27.96
rosemary	889.28 ± 44.23 ^b,c^	18.47 ± 1.09 ^b^	11.53 ± 0.32 ^b^	1393.26 ± 48.06 ^b,c^	19.09 ± 0.74 ^a^	21.28 ± 0.54 ^a^	2282.54	37.56	32.81
sage	6.96 ± 0.29 ^e^	12.93 ± 2.39 ^c,d^	11.84 ± 0.51 ^a,b^	1457.30 ± 21.57 ^b^	16.15 ± 0.59 ^b^	18.43 ± 0.72 ^b^	1464.26	29.08	30.27
thyme	3.58 ± 0.24 ^e^	15.12 ± 1.97 ^b,c^	16.47 ± 1.40 ^a^	1113.25 ± 27.86 ^d^	14.76 ± 1.00 ^c^	18.23 ± 0.41 ^b^	1116.83	29.88	34.70
control	-	6.83 ± 0.06 ^d^	3.05 ± 0.04 ^c^	-	11.09 ± 0.45 ^d^	10.34 ± 0.22 ^f^	-	17.92	13.39

Results are expressed as mean ± standard deviation. Values in the same column followed by different superscript letters are significantly different (*p* < 0.05).

## Data Availability

Not applicable.

## Sample Availability

The samples of compounds are not available from authors.
